# Chemerin and the Gut: From Inflammation to Cancer

**DOI:** 10.3390/biomedicines13112618

**Published:** 2025-10-25

**Authors:** Elvedin Pljakic, Emin Delic, Irfan Corovic, Mladen Maksic, Dusan Radojevic, Isidora Stanisavljevic, Violeta Mladenovic, Tomislav Nikolic, Lejla Suljic, Emina Corovic Licina, Samir Vucelj, Sabir Sagdati, Kemal Corovic, Nebojsa Igrutinovic, Nina Urakovic, Haris Plojovic, Selma Habibovic, Ahmo Habibovic, Dusan Popovic, Milos Nikolic, Marina Jovanovic

**Affiliations:** 1 Center for Molecular Medicine and Stem Cell Research, Faculty of Medical Sciences, University of Kragujevac, Svetozara Markovica 69, 34000 Kragujevac, Serbia; elvedin.pljakic@gmail.com (E.P.); emindelic998@gmail.com (E.D.); isidorastanisavljevic97@gmail.com (I.S.); lejlasuljic990@gmail.com (L.S.); emina.corovic1@gmail.com (E.C.L.); vucelj.samir@hotmail.com (S.V.); ssagdati@gmail.com (S.S.); kemo_dr@hotmail.com (K.C.); selmaredzovic@gmail.com (S.H.); ahmohabibovic@gmail.com (A.H.); 2 General Hospital of Novi Pazar, Generala Zivkovica 1, 36300 Novi Pazar, Serbia; harisplojovic@gmail.com; 3 Department of Internal Medicine, Faculty of Medical Sciences, University of Kragujevac, Svetozara Markovica 69, 34000 Kragujevac, Serbia; asussonicmaster95@gmail.com (M.M.); radojevicdusan@yahoo.com (D.R.); vikicam2004@gmail.com (V.M.); nikolic.s.tomislav@gmail.com (T.N.); shone31084@gmail.com (N.I.); n.urakovic@gmail.com (N.U.); marinna034@gmail.com (M.J.); 4 Clinic for Internal Medicine, University Clinical Centre Kragujevac, Zmaj Jovina 30, 34000 Kragujevac, Serbia; 5 Faculty of Medicine, University of Belgrade, 11000 Belgrade, Serbia; dr.dusan.popovic@gmail.com; 6 Department for Gastroenterology and Hepatology, Clinic for Internal Medicine, University Clinical Hospital Center “Dr Dragisa Misovic-Dedinje”, 11000 Belgrade, Serbia; 7 Department of Pharmacy, Faculty of Medical Sciences, University of Kragujevac, Svetozara Markovica 69, 34000 Kragujevac, Serbia

**Keywords:** chemerin, adipokine, gastrointestinal diseases, biomarker

## Abstract

Chemerin, encoded by the RARRES2 gene, is an adipokine with potent immunometabolic functions mediated through CMKLR1, GPR1, and CCRL2. Its regulation is tissue- and context-dependent, conferring dual protective and pathogenic roles. In the upper GI tract, chemerin facilitates immune tolerance in Barrett’s adenocarcinoma and promotes invasion in esophageal and gastric cancers. In pancreatic disease, it acts as a biomarker of acute and chronic injury, while modulating β-cell function and carcinogenesis. In the liver, chemerin contributes to NAFLD/NASH pathogenesis with both anti-inflammatory and pro-steatotic actions, predicts prognosis in cirrhosis, and demonstrates tumor-suppressive potential in hepatocellular carcinoma. In IBD, chemerin exacerbates colitis via impaired macrophage polarization, yet protects epithelial antimicrobial defense, underscoring its context-specific biology. Collectively, these findings position chemerin as a versatile regulator bridging metabolic dysfunction, inflammation, and gastrointestinal malignancy, and as a potential candidate for biomarker development and therapeutic intervention.

## 1. Introduction

Adipokines are essential mediators that connect metabolic regulation with inflammatory and immune pathways. Among them, chemerin, encoded by RARRES2, has attracted increasing attention for its diverse biological actions. Through its receptors CMKLR1, GPR1, and CCRL2, chemerin influences glucose and lipid metabolism, immune cell recruitment, and tissue remodeling [1]. Its broad tissue distribution, including adipose tissue, liver, and lymphoid organs, positions it as a crucial link between metabolic and inflammatory processes [2]. Chemerin exhibits a context-dependent duality, acting as either an anti-inflammatory or proinflammatory factor depending on physiological and pathological conditions. Elevated circulating levels have been reported in various inflammatory disorders, cardiovascular diseases, and cancers [3,4,5,6], yet its functional outcomes appear highly variable and disease specific.

Within the gastrointestinal tract, adipokines play an important role in maintaining mucosal homeostasis and immune equilibrium [7]. Dysregulated chemerin expression and signaling have been implicated in several gastrointestinal disorders, including chronic inflammation, fibrosis, and malignancy [8,9,10]. However, most studies have focused on individual organs or disease contexts, resulting in a fragmented understanding of chemerin’s overall role in gastrointestinal physiology and pathology.

This review provides a comprehensive and integrative overview of chemerin in gastrointestinal diseases, emphasizing its molecular mechanisms, clinical relevance, and therapeutic potential. By synthesizing findings from experimental and clinical research, it advances beyond existing literature to present chemerin as a dynamic immunometabolic regulator whose context-dependent actions shape gastrointestinal inflammation, tissue repair, and carcinogenesis.

## 2. Chemerin Biology and Receptor Signaling

**Figure 1:**
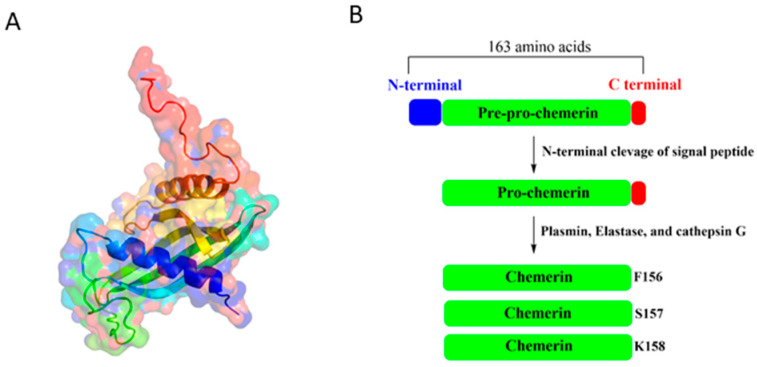
(**A**) 3D structure of chemerin (PDB ID: 8XGM) and (**B**) proteolytic processing of pre-pro-chemerin.

Chemerin, a 137-amino-acid protein encoded by the retinoic acid receptor responder 2 (RARRES2) gene (Figure 1A), is initially synthesized as the 163-amino-acid inactive precursor pre-pro-chemerin [11,12]. This precursor undergoes proteolytic processing, which involves the removal of the N-terminal signal peptide, to produce pro-chemerin with low bioactivity [13]. On the other hand, the removal of the amino acids within the C-terminal segment by plasmin, elastase, and cathepsin G activates chemerin and generates multiple isoforms, including chemerin-K158 (low activity), chemerin-S157 (highest activity), and chemerin-F156 (high activity) (Figure 1B), each exhibiting distinct binding affinities for chemerin receptors [11,14]. Given the crucial role of C-terminal proteolytic processing in regulating chemerin activity, several synthetic peptides, including chemerin-9, chemerin-13 [15], chemerin-15 [16], chemerin-20 [17], chemerin peptide analog CG34 [18], and cyclic peptide-9 [19], have been shown to exhibit bioactivity in various model systems, suggesting their potential utility for experimental or clinical applications.

**Figure 2:**
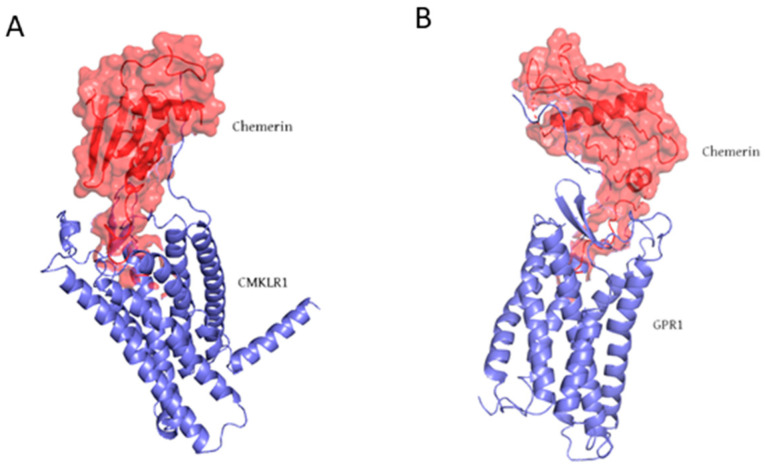
Molecular structure of (**A**) chemerin-CMKLR1 complex (PDB ID: 8ZJG) and (**B**) chemerin-GPR1 complex (PDB ID: 9L3Y). Chemerin is shown as a red surface, while CMKLR1 and GPR1 are shown in purple.

In addition to white adipose tissue and skin, chemerin is abundantly expressed in the lungs, liver, and colon, while lower levels of expression are observed in the small intestine. Circulating chemerin levels are positively correlated with proinflammatory markers, including tumor necrosis factor-alpha (TNF-α), interleukin-6 (IL-6), and C-reactive protein (CRP), suggesting its role as a proinflammatory mediator [13,20]. Aside from its chemotactic function, chemerin, as an adipokine, is strongly linked to adipocyte differentiation, with loss of chemerin expression impairing adipogenesis. The complexity of chemerin biology arises in part from the presence of multiple protein isoforms exhibiting distinct bioactivities within both systemic circulation and local tissue environments. Chemerin exerts its biological effects by interacting with C-C chemokine receptor-like 2 (CCRL2), chemokine-like receptor 1 (CMKLR1) (Figure 2A), and G protein-coupled receptor 1 (GPR1) (Figure 2B) [21,22,23]. CMKLR1, also known as ChemR23, is widely expressed in adipose tissue, as well as in immune and endothelial cells [24]. Interaction with chemerin initiates several intracellular signaling pathways, including Ca^2+^ mobilization, inhibition of cyclic adenosine monophosphate (cAMP) production, and activation of mitogen-activated protein kinases (MAPKs) such as p42/p44 and p38. These downstream events induce a range of cellular responses, most notably chemotaxis, adipogenesis, and regulation of inflammatory pathways, and play a critical role in regulating diverse immunometabolic processes [25,26]. Consequently, this signaling pathway represents an attractive target for novel pharmacological interventions, with multiple candidate drugs already identified and progressing through various stages of clinical development [23].

**Figure 3:**
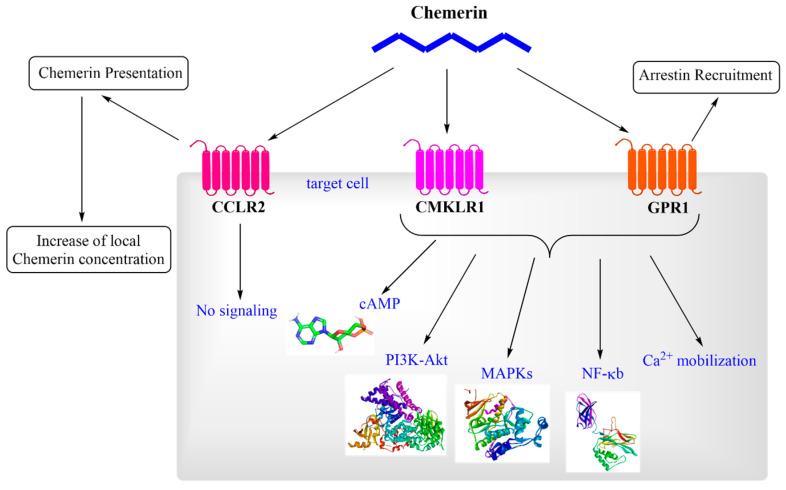
Molecular pathways activated by chemerin. The abbreviations are as follows: CCRL2 (C-C chemokine receptor-like 2), CMKLR1 (chemokine-like receptor 1), GPR1 (G protein-coupled receptor 1), MAPK (mitogen-activated protein kinase), cAMP (cyclic adenosine monophosphate), NF-κB (nuclear factor kappa-light-chain-enhancer of activated B cells), and PI3K-Akt (phosphatidylinositol 3′-kinase-Akt).

In contrast, CCRL2 functions as a non-signaling “decoy” receptor, binding chemerin without triggering downstream signaling events. Specifically, CCRL2 facilitates the presentation of chemerin to neighboring CMKLR1-expressing cells, thereby increasing local chemerin availability and amplifying CMKLR1-dependent responses [26]. CCRL2 is expressed in leukocytes, endothelial cells, and various other immune cell types, highlighting its role in modulating chemerin activity during inflammatory responses [27]. GPR1 shares structural homology with CMKLR1, but exhibits distinct functional properties. Although GPR1 binds chemerin with high affinity, it elicits only minimal Ca^2+^ mobilization compared to CMKLR1. Instead of classical G protein-dependent signaling, GPR1 primarily engages arrestin-mediated pathways. This mechanism leads to prolonged cellular outcomes such as receptor internalization and activation of alternative cascades, including MAPKs, which regulate long-term cellular functions. GPR1 expression is most prominent in the central nervous system and selected peripheral tissues, suggesting potential roles in neuroendocrine regulation and metabolic control. Although its precise physiological and pathological significance remains to be fully elucidated, the unique expression profile of GPR1 indicates functions distinct from those of CMKLR1 and CCRL2 [26,28,29]. Figure 3 provides a summarized overview of chemerin signaling, illustrating its interactions with receptors and downstream effects.

## 3. Chemerin in the Upper Gastrointestinal Tract

### 3.1. Chemerin in Esophageal Pathology

Chemerin expression is detectable in healthy esophageal epithelium, where it may function as a rapid-response mediator for the recruitment of effector immune cells, paralleling its role in other barrier tissues [30]. In disease states, chemerin expression is dynamically regulated and exerts a pivotal influence on esophageal pathology. In esophageal squamous cell carcinoma, chemerin acts as a stromal factor overexpressed by cancer-associated myofibroblasts, recruiting CMKLR1-expressing mesenchymal stromal cells to the tumor microenvironment, thereby fostering a pro-invasive and pro-angiogenic stroma [31]. Additionally, chemerin-stimulated mesenchymal stromal cells display enhanced secretion and adhesion, which in turn augments myofibroblast adhesion, migration, and proliferation [32]. Moreover, both patient-derived and commercial (OE21) esophageal squamous cell carcinoma cell lines express CMKLR1, and chemerin secreted by cancer-associated myofibroblasts drives their migration, invasion, and proliferation. It was further demonstrated that in the OE21 cell line, chemerin enhances invasion by inducing matrix metalloproteinase activity through PKC and MAPK activation. Notably, inhibiting chemerin actions through neutralization, small interfering RNA (siRNA) knockdown, or ChemR23 antagonism (CCX832) reduces invasion in both Boyden chamber and organotypic models, underscoring chemerin’s direct role in promoting tumor aggressiveness [33].

Regarding its role in esophageal adenocarcinoma, chemerin expression increases progressively from metaplastic to dysplastic and invasive stages during the transition from Barrett’s esophagus to adenocarcinoma, likely serving as a chemoattractant for myeloid dendritic cells that drive regulatory T-cell differentiation and suppress anti-tumor immunity, thereby facilitating malignant progression [30]. In patients with esophageal cancer and metabolic syndrome, an expedited surgical protocol was associated with greater reductions in circulating chemerin levels than the standard protocol, alongside decreases in inflammatory markers such as TNF-α, high-sensitivity CRP, and leptin; these changes coincided with improved lipid metabolism, nutritional recovery, and quality-of-life scores [34].

**Figure 4:**
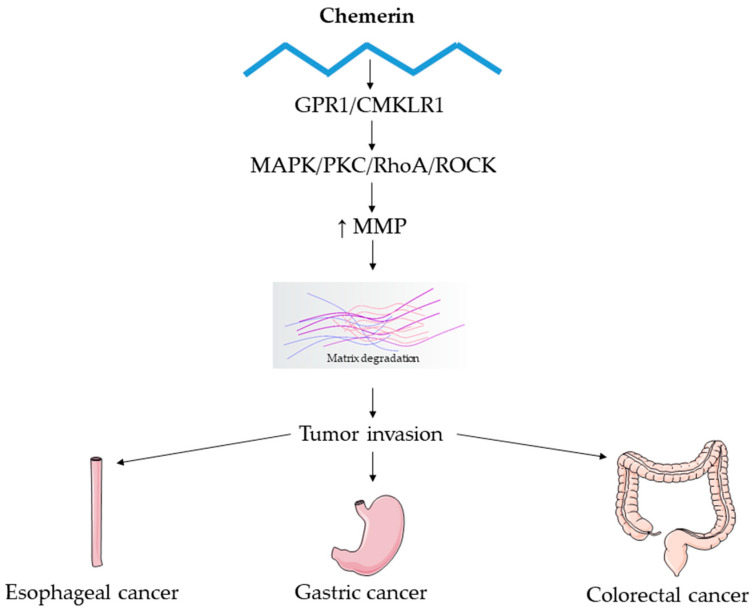
Shared molecular mechanisms in esophageal, gastric, and colorectal cancers associated with chemerin signaling. This figure illustrates the shared molecular mechanisms underlying esophageal, gastric, and colorectal carcinogenesis, emphasizing the role of chemerin-mediated modulation of MMPs and their contribution to extracellular matrix degradation, tissue remodeling, and tumor invasion.

Overall, available data suggest that chemerin plays an aggressive role in esophageal cancer pathology (Figure 4). However, evidence is very scarce, primarily derived from in vitro studies and a single patient cohort, and further mechanistic and translational research is needed to clarify its receptor-specific effects and clinical relevance.

### 3.2. Chemerin in Gastric Pathology

Gastric cancer continues to be a significant global health issue, with nearly one million new diagnoses each year and over 650,000 deaths attributed to the disease. Recognized risk factors encompass *Helicobacter pylori* infection, dietary patterns, obesity, smoking, and genetic factors, highlighting the complex nature of gastric cancer development [35]. Adipokines are recognized as important modulators of gastric physiology and pathology, influencing metabolic signaling, mucosal inflammation, and tumor progression within the stomach [7]. Early evidence from Wang et al. [36] demonstrated elevated serum chemerin levels in gastric cancer patients (n = 36), detectable even at early stages and further increased in advanced and non-intestinal subtypes. In a larger cohort (n = 196), Zhang et al. [37] confirmed significantly higher preoperative plasma chemerin concentrations in gastric cancer patients compared with healthy controls, correlating with tumor stage, lymph node involvement, distant metastasis, peritoneal spread, and poor prognosis. Chemerin was further identified as an independent prognostic marker for both overall and disease-free survival, showing strong predictive power for 5-year outcomes.

These clinical observations prompted further mechanistic studies demonstrating that chemerin promotes epithelial–mesenchymal transition (EMT), migration, and invasion of gastric adenocarcinoma (AGS) cell lines via both GPR1 and CMKLR1, through activation of the mitogen-activated protein kinase (MAPK), Ras homolog family member A/Rho-associated protein kinase (RhoA/ROCK), and protein kinase C (PKC) signaling pathways. This signaling cascade upregulates vascular endothelial growth factor (VEGF), matrix metalloproteinase-7 (MMP-7), and interleukin-6 (IL-6), while downregulating tissue inhibitors of metalloproteinases-1 and -2 (TIMP-1 and TIMP-2), thereby enhancing matrix metalloproteinase (MMP) activity and extracellular matrix degradation [36,38,39]. These effects occur without influencing cell proliferation, underscoring chemerin’s role as a key driver of invasiveness in gastric cancer. Moreover, Kumar et al. [39] identified cancer-associated myofibroblasts as the main source of chemerin within the gastric cancer microenvironment, confirming their own findings in esophageal squamous cell carcinoma, where these cells were likewise shown to represent the principal chemerin source.

Furthermore, *H. pylori* infection has been shown to increase chemerin expression in gastric epithelial cell lines (KATO III and AGS) and to elevate its expression in gastric tissue from patients with chronic ulcers, suggesting that infection-driven inflammation may potentiate chemerin-mediated tumorigenic signaling [40].

However, the current evidence remains limited. Most findings derive from a small number of in vitro studies, without further replication or expansion of the proposed mechanisms, and lack in vivo validation. Clinical data are also restricted to single measurements, without evaluation of postoperative or longitudinal changes. Future large-scale, mechanistically integrated, and reproducible studies are essential to confirm chemerin’s biological significance, prognostic value, and therapeutic potential in gastric cancer.

## 4. Chemerin in Pancreas Pathology

Chemerin and its receptor CMKLR1 are expressed in adipose tissue, immune cells, and pancreatic β-cells, linking metabolic and inflammatory pathways to pancreatic physiology [41]. In clinical settings, elevated pre-procedural chemerin, together with insulin resistance, independently predicted the risk of post-ERCP pancreatitis [42], suggesting that endogenous chemerin elevation reflects metabolic-inflammatory susceptibility, likely driven by adipose-derived inflammation. Similarly, Yin et al. [43] found higher serum chemerin levels in hyperlipidemia-induced pancreatitis, correlating with disease severity and suggesting its role as a biomarker of inflammatory burden. In contrast, in a rat model of caerulein-induced pancreatitis, chemerin pre-administration markedly reduced pancreatic injury, decreasing edema, serum amylase, and TNF-α levels. In vitro, it suppressed TNF-α expression and shifted NF-κB signaling toward non-canonical p50/p50/Bcl-3 complexes, limiting proinflammatory gene transcription [44]. Notably, these findings indicate that chemerin’s function is context-dependent: endogenous elevation reflects a predisposed and reactive metabolic–inflammatory state, whereas controlled exogenous activation exerts anti-inflammatory and cytoprotective effects. It cannot be excluded that this endogenous rise also represents a counter-regulatory or compensatory mechanism aimed at limiting excessive tissue injury, which would be consistent with experimental observations. Differences in timing, source (endogenous vs. exogenous), and study model (clinical vs. experimental) likely account for these divergent outcomes.

Regarding chemerin’s role in chronic pancreatitis, Adrych et al. [45] first reported increased serum chemerin levels in patients with chronic pancreatitis, independent of diabetes or BMI reduction, suggesting that chemerin reflects local inflammatory and fibrogenic activity rather than adiposity. Its correlation with profibrotic cytokines such as PDGF-BB and TGF-β1 supports a role in pancreatic stellate cell activation and fibrosis progression. Kiczmer et al. [46] confirmed persistent chemerin elevation in chronic pancreatitis and markedly elevated levels in pancreatic ductal adenocarcinoma, with concentrations unrelated to BMI, implying disease-specific regulation. Mechanistically, chemerin may promote angiogenesis and stromal remodeling through CMKLR1-dependent MAPK/AKT signaling. Conversely, Tu et al. [47] found markedly reduced chemerin levels in pancreatogenic diabetes (type 3c), associated with β-cell dysfunction and reduced GLUT2 and PDX1 expression. Pharmacological CMKLR1 activation with the chemerin-9 agonist restored β-cell transcriptional programs and improved glucose tolerance.

Together, these findings highlight chemerin’s context-dependent duality: elevated in chronic inflammatory and neoplastic states, potentially reflecting reactive or compensatory fibrogenic activity, but decreased in advanced endocrine failure, where its deficiency becomes maladaptive (Table 1). Variability in disease stage, cellular source, and study design likely accounts for these discrepancies. However, current evidence remains limited. Most studies rely on small cohorts or in vitro and short-term experimental models, without serial measurements or receptor-level characterization. Future research should include longitudinal human studies to determine whether dynamic changes in chemerin levels parallel disease progression or resolution and to clarify whether chemerin acts predominantly as a marker of disease severity or a modulator of protective immune regulation in pancreatic diseases.

## 5. Chemerin in Liver Diseases

### 5.1. Chemerin and Fatty Liver Disease

#### 5.1.1. Experimental Data

**Figure 5:**
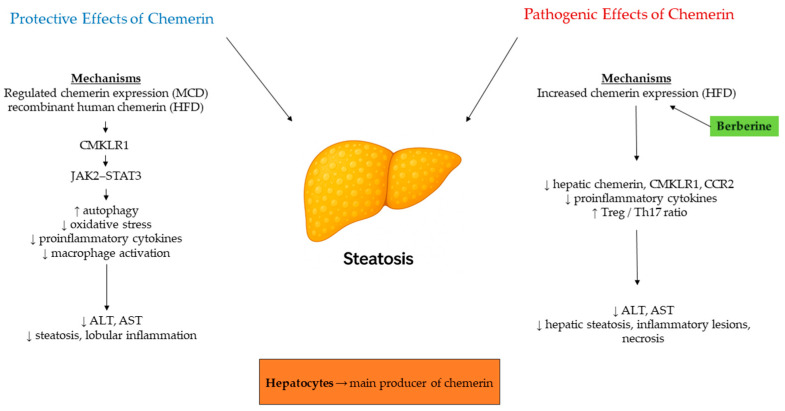
Chemerin in experimental NAFLD/NASH. Hepatocytes are the main hepatic source, with increased expression under steatotic and inflammatory conditions. Experimental models show that balanced chemerin/CMKLR1 signaling reduces inflammation, oxidative stress, and improves insulin sensitivity, whereas berberine administration attenuates hepatic steatosis and necrosis by reducing chemerin, CMKLR1, and CCR2 expression. Arrows indicate direction of change (↑ increase, ↓ decrease).

Chemerin is an adipokine that plays a significant role in the differentiation of adipocytes, the maintenance of glucose levels, and the regulation of inflammation [48]. Its strong connection to obesity and metabolic syndrome, two major risk factors for nonalcoholic fatty liver disease (NAFLD), has established chemerin as a possible biomarker and mediator in the mechanisms behind NAFLD and its advanced form, nonalcoholic steatohepatitis (NASH) [49]. The recent shift in terminology from NAFLD to metabolic dysfunction-associated steatotic liver disease (MASLD) has sharpened the focus on the metabolic factors underlying the condition, further emphasizing the importance of adipokines, such as chemerin [50,51]. Experimental models have provided essential insights into the complex role of chemerin in the development of fatty liver disease. Experimental evidence establishes hepatocytes as the principal hepatic source of chemerin, displaying markedly higher expression than other liver cell types. In human NAFLD and NASH, hepatic chemerin mRNA is consistently elevated, although corresponding protein levels show variable patterns, suggesting post-transcriptional regulation. In mouse models, diet-dependent differences were observed: the high-fat diet and ob/ob mice, which model simple hepatic steatosis, showed increased hepatic chemerin mRNA expression, while the Paigen and methionine–choline-deficient (MCD) diets, both inducing NASH with inflammation and fibrosis, led to increased hepatic and, partially, circulating chemerin [52]. Functionally, hepatocyte-specific chemerin overexpression in MCD-fed mice increased hepatic and serum chemerin but attenuated oxidative stress and macrophage-driven inflammation, reducing IL-6, CCL2, and osteopontin production [49]. Similarly, recombinant human chemerin treatment in high-fat diet mice alleviated steatosis and inflammation, improved insulin sensitivity, and decreased ALT/AST levels via CMKLR1-dependent JAK2–STAT3 activation, which enhanced autophagy and reduced oxidative stress [53,54]. In contrast, treatment with berberine notably improved NAS scores and diminished hepatocellular injury in rats with HFD-induced NASH. Simultaneously, it downregulated hepatic expression of chemerin, CMKLR1, and CCR2, while reducing proinflammatory cytokines. Beyond the hepatocellular benefits, berberine also reestablished the Treg/Th17 balance, indicating that chemerin/CMKLR1 signaling potentially plays a role in modulating both innate immunity and inflammation, as well as reshaping adaptive immune balance within the hepatic microenvironment [55]. Collectively, these experimental results emphasize the dual nature of chemerin in NAFLD/NASH (Figure 5). When properly regulated, chemerin exhibits hepatoprotective effects by suppressing inflammation, promoting autophagy, and reducing oxidative stress. Conversely, in proinflammatory metabolic conditions, excessive chemerin/CMKLR1 activity may exacerbate steatohepatitis, and its inhibition, as demonstrated by berberine, can yield beneficial effects. This context-sensitive behavior highlights the complexity of chemerin as a potential therapeutic target and a biomarker for disease stratification.

#### 5.1.2. Clinical Data

Clinical evidence consistently supports a link between circulating chemerin and systemic metabolic dysfunction. Elevated chemerin levels correlate with BMI, total body fat, dyslipidemia, and impaired glucose metabolism, aligning with its proposed role as a biomarker of metabolic imbalance in NAFLD [51]. Multiple studies show increased serum chemerin in NAFLD and particularly in NASH, where it associates more closely with hepatocellular injury and inflammation than with fibrosis [56,57]. Therapeutic interventions such as L-carnitine and metformin have been shown to reduce chemerin levels alongside improvements in liver enzymes and insulin sensitivity, further reinforcing this metabolic association [58,59]. Moreover, in diabetic cohorts, Zhang et al. [60] identified chemerin as an independent risk factor for NAFLD, with levels increasing in parallel with steatosis severity and closely associated with obesity, dyslipidemia, and HOMA-IR. Comprehensive population-based research by Levin et al. [61] demonstrated strong associations between chemerin and hepatic steatosis, as measured by magnetic resonance imaging (MRI), revealing a linear relationship in men and a U-shaped pattern in women, unaffected by other health complications.

At the tissue level, chemerin regulation shows considerable heterogeneity. Pohl et al. [62] reported reduced hepatic chemerin mRNA in NASH, inversely correlated with inflammation and fibrosis, indicating an extrahepatic source of circulating chemerin. Kajor et al. [63] further demonstrated that adipose tissue, rather than the liver, is the main contributor to systemic chemerin in morbid obesity. Similarly, Bekaert et al. [64] observed that lower chemerin expression in visceral adipose tissue was inversely associated with steatosis and NAFLD activity, suggesting impaired adipose–liver signaling in metabolic dysfunction. Together, these findings emphasize the tissue-specific and context-dependent regulation of chemerin and highlight the importance of considering adipose contributions when evaluating its role in metabolic liver disease. A recent meta-analysis confirms that circulating chemerin is elevated in MAFLD and NAFLD but not consistently in NASH, indicating that chemerin reflects metabolic dysfunction and steatosis more reliably than histological severity [65].

Collectively, these findings (Table 2) identify serum chemerin as a potential biomarker of metabolic dysfunction and hepatic steatosis, while its relationship with histological severity remains inconsistent. However, a key limitation across clinical studies is the discrepancy between elevated circulating chemerin and reduced hepatic expression reported in some tissue analyses. This divergence suggests that circulating chemerin may primarily originate from adipose tissue rather than the liver, reflecting systemic rather than hepatic metabolic activity.

### 5.2. Chemerin in Liver Cirrhosis

**Figure 6:**
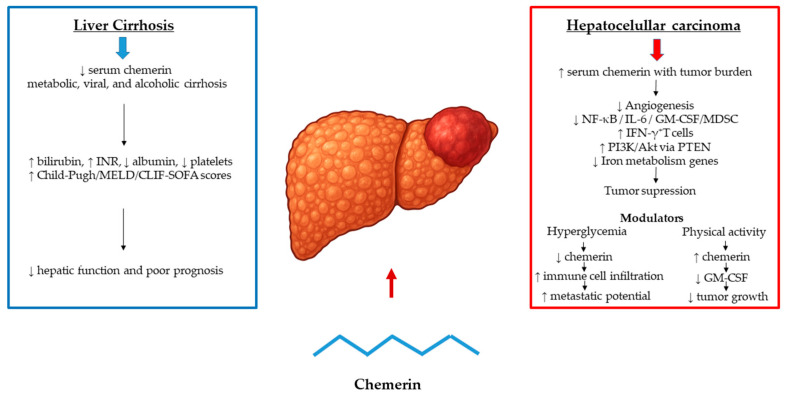
Chemerin at the Crossroads of Cirrhosis and HCC. Chemerin levels decline with cirrhosis severity, correlating with impaired hepatic function and predicting short-term mortality. In HCC, circulating chemerin paradoxically rise with tumor burden supporting tumor-suppressive roles. Arrows indicate direction of change (↑ increase, ↓ decrease).

Chemerin, an adipokine with key immunometabolic functions, is mainly produced in the liver and adipose tissue and has been increasingly investigated as an indicator of hepatic functional status in cirrhosis. Early clinical investigations by Eisinger et al. [66] demonstrated that hepatic chemerin secretion declines with worsening liver function, with serum levels decreasing across Child–Pugh classes and reaching their lowest in Child C patients. This reduction reflected impaired coagulation rather than hepatic gene expression, indicating that circulating chemerin mirrors residual hepatic function rather than synthetic capacity alone. Horn et al. [67] further showed that low chemerin levels in decompensated cirrhosis correlate with bilirubin, albumin, INR, platelet count, and disease severity scores (Child–Pugh, MELD, CLIF-SOFA), with concentrations below 87 ng/mL independently predicting 28-day mortality or transplantation. Similarly, Peschel et al. [68] reported that chemerin correlates with hepatic dysfunction and fibrosis in chronic hepatitis C, independent of viral load or genotype, and remains unchanged after antiviral therapy, again reflecting liver function rather than viral activity. In alcoholic cirrhosis, Prystupa et al. [69] confirmed a progressive decline across Child–Pugh stages, strongly linked to bilirubin, INR, and albumin. Collectively, these studies identify low circulating chemerin as a consistent feature of advanced cirrhosis, reflecting hepatic dysfunction and portal hypertension, and suggesting potential, but still preliminary utility for risk assessment in end-stage liver disease (Figure 6).

### 5.3. Chemerin in Liver Cancer

Hepatocellular carcinoma (HCC), the most common primary liver cancer, typically develops in cirrhotic livers and remains a major cause of cancer-related mortality worldwide [70]. Among various adipokines implicated in hepatocarcinogenesis, chemerin has attracted attention due to its immunometabolic, angiogenic, and metabolic regulatory roles [71,72]. Yet, its function in HCC appears to be context-dependent. Circulating chemerin levels are reduced in cirrhosis and decline with worsening hepatic function, but paradoxically increase with tumor burden in HCC, sometimes approaching concentrations seen in healthy controls—likely reflecting compensatory release from non-tumorous tissue. [70]. Moreover, in the NASH-related HCC mouse model, chemerin expression and receptor activation remain unchanged between tumor-bearing and control groups, despite the presence of liver tumors suggesting that high endogenous chemerin is not sufficient to prevent hepatocarcinogenesis [73]. Overexpression of the active chemerin-156 isoform reduced the number of small lesions but failed to limit established tumor growth or alter inflammatory and fibrotic gene expression [49]. Similarly, in diabetic HCC models, hyperglycemia-induced suppression of chemerin was linked to a more immunosuppressive tumor microenvironment, reduced immune cell infiltration, and enhanced metastatic potential [74].

Conversely, growing evidence supports a tumor-suppressive role for chemerin, particularly in early tumorigenesis. Lower serum and intratumoral chemerin levels in HCC patients correlate with poorer prognosis [70], while chemerin overexpression in mice inhibits angiogenesis, enhances immune activation, and suppresses NF-κB, IL-6, and GM-CSF signaling, leading to reduced myeloid-derived suppressor cell accumulation and increased IFN-γ–producing T cells [75,76]. Chemerin also modulates PI3K/Akt signaling via PTEN-dependent inhibition [77,78] and induces cell-cycle arrest in hepatoma cells through downregulation of iron metabolism genes and activation of the p53/p27/p21 axis [79]. These findings suggest that chemerin interferes with multiple tumor-promoting mechanisms, including proliferation, angiogenesis, and immune evasion. The heterogeneity of these findings likely reflects differences in disease etiology and population characteristics. While decreased chemerin levels have been reported in HBV-associated HCC in Asian cohorts, increased expression in NAFLD-related and idiopathic HCC has been noted in European studies, despite consistent CMKLR1 downregulation across tumor types, potentially limiting downstream signaling [80]. Environmental and systemic factors further influence this axis; for example, voluntary exercise enhances chemerin secretion from brown adipose tissue, reducing tumor growth through GM-CSF suppression and antiproliferative effects in HCC models [81]. Notably, in HCC, but not in colorectal cancer liver metastasis, adiponectin correlated with chemerin levels, suggesting a liver-specific pathogenic role [82].

Collectively, these observations suggest that chemerin exerts multifaceted, context-dependent effects in hepatocarcinogenesis, intersecting metabolic, immune, and angiogenic pathways, while its clinical utility remains to be fully defined.

## 6. Chemerin in Lower Gastrointestinal Tract Pathology

### 6.1. Chemerin in IBD

#### 6.1.1. Experimental Data

Inflammatory bowel disease (IBD), encompassing Crohn’s disease (CD) and ulcerative colitis (UC), is a chronic intestinal disorder driven by genetic, microbial, environmental, and immune factors [83]. Visceral adipose tissue actively contributes to mucosal inflammation through adipokine release, positioning chemerin as a potential immunometabolic mediator in IBD [6,84,85]. In dextran sodium sulfate (DSS)-induced colitis, exogenous chemerin aggravated disease severity, causing greater weight loss, mucosal damage, and mortality, while elevating IL-6, TNF-α, and IFN-γ levels. Mechanistically, chemerin disrupted IL-4/STAT6-dependent M2 macrophage polarization, reducing Arg-1, Ym1, FIZZ1, and IL-10 expression and impairing inflammation resolution. Neutralizing chemerin improved mucosal healing, underscoring its pathogenic contribution [86]. Chemerin and its receptor CMKLR1 are upregulated in colitis, particularly in the distal colon, with bioactive chemerin levels remaining high despite overall serum decline. CMKLR1-deficient mice displayed delayed disease onset and altered cytokine responses, but the final severity of the disease was comparable, suggesting that chemerin signaling primarily influences early inflammation rather than disease progression [87]. Therapeutic inhibition of chemerin, such as with cornelian cherry extract, attenuated trinitrobenzene sulfonic acid (TNBS) colitis by reducing TNF-α, IL-17, and chemerin, strengthening epithelial barrier integrity and limiting pathogenic *E. coli* [88]. Conversely, chemerin appears protective at the epithelial level: its absence, or loss of CMKLR1, increases susceptibility to neutrophilic colitis and colitis-associated cancer by downregulating lactoperoxidase (LPO), an enzyme essential for microbial control. Restoration of LPO reverses this phenotype, highlighting the chemerin-CMKLR1-LPO axis in mucosal defense [89].

Collectively, these insights position chemerin as a regulator that exerts context-dependent effects in experimental IBD: harmful when it obstructs macrophage-mediated inflammation resolution, but beneficial when it supports epithelial antimicrobial activity and microbial stability. This complexity underscores the potential of chemerin as both a marker of disease activity and a therapeutic target.

#### 6.1.2. Clinical Data

Although research on chemerin in IBD remains limited, current evidence indicates dysregulated expression with possible clinical relevance. Weigert et al. [90] first reported elevated serum chemerin levels in both Crohn’s disease and ulcerative colitis, particularly among men with Crohn’s disease, with gender-specific links to disease activity and duration. In contrast, Waluga et al. [91] found no significant differences compared to controls, suggesting heterogeneity across cohorts. Subsequent studies provided functional context: Terzoudis et al. [92] associated higher serum chemerin with osteoporosis in IBD, implying a role in extraintestinal metabolic complications, while Sochal et al. [93] observed increased levels during active disease, especially in ulcerative colitis, and normalization following anti-TNF therapy, suggesting responsiveness to inflammatory control. Beyond serum findings, Gunawan et al. [94] detected measurable urinary chemerin, markedly elevated in patients with high fecal calprotectin, introducing a potential noninvasive indicator of intestinal inflammation. A recent meta-analysis including 2717 participants (799 with Crohn’s disease, 520 with ulcerative colitis, and 609 healthy controls) demonstrated that circulating chemerin levels are significantly elevated in inflammatory bowel disease, especially during active phases. Based on these results, chemerin has been proposed as a potential biomarker for the diagnosis and monitoring of disease activity, reflecting its involvement in intestinal immune regulation and chronic inflammation [8].

Collectively, these studies highlight chemerin’s inconsistent systemic behavior but suggest its potential utility in reflecting disease activity, therapeutic response, and metabolic comorbidity in IBD.

### 6.2. Chemerin and Irritable Bowel Syndrome

Irritable bowel syndrome (IBS) is a prevalent functional gastrointestinal disorder marked by recurrent abdominal pain and altered bowel habits, affecting up to 10% of the population. Its pathophysiology involves gut–brain axis dysregulation, microbial imbalance, immune alterations, and psychosocial stressors [95]. Increasing evidence also links IBS to metabolic syndrome, suggesting overlapping metabolic and inflammatory pathways [96]. Given chemerin’s established role in metabolic dysfunction [6], it has been proposed as a potential mediator connecting metabolic and intestinal disturbances, though dedicated research remains scarce. Recent studies have begun to explore this association. Baram et al. [97] reported elevated circulating chemerin levels in adults with IBS, particularly in diarrhea-predominant cases, correlating with symptom severity, abdominal pain, bloating, and psychosocial stress. Similarly, Roczniak et al. [98] found higher serum chemerin and lower omentin-1 in children with IBS, a pattern linked to insulin resistance and dyslipidemia, which persisted after metabolic adjustment. Notably, chemerin levels above 232.8 ng/mL demonstrated limited sensitivity (39%) but high specificity (87%) for distinguishing IBS patients from healthy individuals.

Collectively, these findings (Table 3) suggest that chemerin may represent a functional link between metabolic imbalance and intestinal dysregulation in IBS, with potential relevance for disease characterization rather than established diagnostic use. Further longitudinal studies are needed to clarify its mechanistic and clinical implications.

### 6.3. Chemerin in Colorectal Cancer (CRC)

Worldwide, CRC represents one of the most prevalent cancers and remains a leading cause of cancer-associated mortality [99]. Growing evidence suggests that chemerin has a complex role in colorectal carcinogenesis, influencing early adenoma development, cancer risk, disease progression, and survival outcomes. In a case–control study, Yagi et al. [100] reported higher serum chemerin levels in men with colorectal adenomas, identifying it as an independent predictor of adenoma presence, while the EPIC-Potsdam cohort linked elevated baseline chemerin with an increased risk of colorectal cancer, particularly in the proximal colon, independent of BMI and inflammatory markers [101]. These findings suggest that chemerin may contribute to the adenoma-carcinoma sequence and CRC susceptibility.

In established disease, circulating chemerin is consistently elevated and correlates with TNM stage, CRP, CEA, CA19-9, and fibrinogen, reflecting both tumor burden and systemic inflammation [102,103]. Although early clinical studies suggested high diagnostic accuracy, these estimates likely overstate its predictive capacity. Beyond diagnosis, elevated chemerin in CRC survivors has been associated with greater fatigue and reduced quality of life [104], supporting its role as a marker of persistent systemic inflammation. Importantly, Waniczek et al. [105] extended these findings to saliva, demonstrating markedly elevated salivary chemerin levels in CRC patients with 99% specificity and 100% sensitivity at a cut-off of 231.24 ng/mL, though the small sample size limited the analysis and requires independent validation.

Mechanistically, recent studies reveal that chemerin modulates tumor angiogenesis, extracellular matrix remodeling, and immune interactions. Increased CMKLR1 and MMP-9 expression in tumor tissue, along with links to VCAM-1, indicate that chemerin facilitates angiogenic and matrix-remodeling processes [106]. Kiczmer et al. further demonstrated that higher CMKLR1 expression is associated with lower vascularity and reduced tumor budding, implying that CMKLR1 may have a multifaceted role in colorectal cancer pathogenesis by regulating tumor architecture and shaping peritumoral immune infiltration [107]. Experimental activation of CMKLR1 with the chemerin analog CG34 enhanced colony formation, vascularization, and tumor growth in CRC models [18], while genetic and epigenetic analyses identified chemerin-associated regulatory loci within known CRC susceptibility regions [108]. Integrating single-cell and spatial transcriptomics, Qi et al. [109] uncovered a chemerin-mediated interaction between FAP^+^ fibroblasts and SPP1^+^ macrophages, fostering a desmoplastic, immunosuppressive tumor microenvironment linked to T-cell exclusion, poor survival, and resistance to anti–PD-L1 therapy.

Overall, available evidence indicates that chemerin-CMKLR1 signaling is involved in multiple stages of colorectal carcinogenesis (Figure 4), linking metabolic, inflammatory, and stromal pathways. However, its precise clinical relevance and therapeutic implications remain to be fully clarified.

## 7. Research Gaps and Future Perspectives

Although chemerin has been increasingly recognized as an important regulator of gastrointestinal physiology and pathology, several critical research gaps persist. Most existing evidence is derived from observational or in vitro studies, while mechanistic in vivo, longitudinal, and organ-specific models capable of clarifying receptor-dependent pathways across different regions of the gastrointestinal tract remain limited. The isoform-dependent activity of chemerin and its distinct signaling through CMKLR1, GPR1, and CCRL2 receptors [11,14] are still poorly characterized. Furthermore, the temporal and spatial dynamics of chemerin expression during the transition from acute to chronic inflammation, and ultimately to malignancy, have not been systematically investigated.

The relative contributions of circulating versus tissue-specific chemerin also remain unclear, particularly regarding its diagnostic, prognostic, and predictive value for disease activity and therapeutic response. Moreover, the lack of standardization in chemerin measurement methodologies, including variability in assay types, sample sources, and detection thresholds, limits the comparability of results across studies and hampers reliable biomarker validation. Importantly, many available studies have not adequately controlled for the confounding influence of obesity, a major determinant of circulating chemerin levels and a well-established risk factor for gastrointestinal disorders, which may obscure true disease-specific associations. Additionally, the interaction between chemerin and the gut microbiota remains largely unexplored, despite growing evidence that microbial metabolites can modulate adipokine signaling and mucosal immune responses [110].

Although chemerin and its receptors have been extensively implicated in inflammation, metabolic regulation, and tissue homeostasis [4], pharmacological manipulation of this axis remains in its infancy. The field still lacks clinically tested agonists or antagonists capable of selectively modulating chemerin signaling in humans. Most current data originate from preclinical studies employing peptide mimetics (e.g., chemerin-9, CG34) [47,107] or experimental small-molecule CMKLR1 (ChemR23) inhibitors (e.g., CCX832) [33], whose stability, receptor selectivity, and pharmacokinetic profiles remain suboptimal. Moreover, the dual and context-dependent role of chemerin adds further complexity to therapeutic targeting. Recent advances, including the resolution of the CMKLR1 crystal structure [23,111] and the development of agonistic monoclonal antibodies (e.g., OSE-230/ABBV-230) [112] that promote inflammatory resolution, highlight promising directions for translational research. However, several key questions remain unresolved: which receptor subtypes mediate beneficial versus deleterious effects, how proteolytic processing alters receptor bias, and under which temporal or tissue conditions therapeutic intervention would be both safe and effective.

Future research should integrate structural pharmacology, spatial transcriptomics, multi-omics profiling, and disease-stage-specific modeling to better define actionable therapeutic windows for chemerin modulation. Furthermore, longitudinal clinical cohorts, organoid-based disease models, and interventional studies are required to establish causal relationships, validate therapeutic efficacy, and translate preclinical discoveries into clinical applications.

## 8. Limitations

This review is limited by the heterogeneity of existing studies, including variations in patient populations, disease models, detection methods, and reporting of chemerin isoforms. The inclusion of both experimental and clinical data introduces interpretative complexity, as many studies lack standardized outcome measures. Furthermore, due to the narrative nature of this review, quantitative synthesis and meta-analytic validation were not feasible. Finally, publication bias and the predominance of small, single-center studies may have influenced the overall interpretation of chemerin’s role in gastrointestinal diseases.

## 9. Conclusions

Chemerin has emerged as a pivotal link between metabolism, inflammation, and gastrointestinal pathology. Evidence across studies indicates that altered chemerin signaling contributes to mucosal immune imbalance, fibrosis, and tumor progression within the gastrointestinal tract. However, its dual and context-dependent underscore the complexity of its biological actions. A deeper understanding of receptor-specific mechanisms, isoform diversity, and metabolic confounders such as obesity is essential to clarify chemerin’s precise role in gastrointestinal disease. Ultimately, advancing standardized methodologies, integrative multi-omics analyses, and translational research into receptor-selective agonists or antagonists will be critical to unlock chemerin’s diagnostic and therapeutic potential.

## Figures and Tables

**Table 1:** Key studies on chemerin in pancreatic pathology.

First Author/Type of Study	Context/Population	Key Findings
Köksal et al. [42] Clinical (prospective)	PEP	↑ pre-procedural serum chemerin predicts PEP risk
Yin et al. [43] Clinical (cohort)	HLAP	↑ serum chemerin in severe HLAP
Jaworek et al. [44] Experimental (rat model, in vitro)	Caerulein-induced acute pancreatitis	Exogenous chemerin ↓ acute pancreatitis severity
Kiczmer et al. [46] Clinical (case–control)	Chronic pancreatitis Pancreatic cancer	↑ serum chemerin ↑↑ serum chemerin
Tu et al. [47] Clinical (case–control) + experimental (murine) study	Pancreatogenic diabetes (type 3c)	↓ serum chemerin; chemerin agonist ↑ function of β-cells

Arrows indicate direction of change (↑ increase, ↓ decrease).

**Table 2:** Clinical Studies on Chemerin and MAFLD/NAFLD/NASH.

First Author/Study Type	Context/Population	Key Findings
Hamza et al. [58] Interventional	Obese children + NAFLD + L-carnitine	↑ serum chemerin correlates with NAFLD severity; decreased after L-carnitine treatment
Zhang Et Al. [60] Cross-sectional	Type 2 diabetes mellitus (NAFLD vs. non-NAFLD)	Chemerin independent risk factor for NAFLD
Levin Et Al. [61] Population-based study	General population (SHIP-TREND; metabolic/liver disease context)	Chemerin is associated with liver fat; independent of comorbidities
Pohl et al. [62] Cross-sectional	NASH	↓ hepatic chemerin mRNA in NASH
Kajor et al. [63] Cross-sectional	Morbidly obese women + NAFLD	No correlation between serum and hepatic chemerin; adipose tissue likely main source
Ren et al. [65] Systematic review and meta-analysis	MAFLD/NAFLD/NASH 17 studies, 2580 participants	↑ serum chemerin in MAFLD/NAFLD; No difference in NASH, fibrosis, inflammation

Arrows indicate direction of change (↑ increase, ↓ decrease).

**Table 3:** Clinical studies investigating chemerin in IBD and IBS.

First Author/Study Type	Context/Population	Key Findings
Weigert et al. [90] Cross-sectional observational	CD + UC	↑ chemerin in both CD and UC; sex- and phenotype-specific patterns
Waluga et al. [91] Prospective observational	CD + UC (before/after steroids/ azathioprine therapy)	No change vs. controls; not therapy- or activity-related
Terzoudis et al. [92] Case-control	CD + UC	↑ in IBD; predicts osteoporosis
Sochal et al. [93] Prospective observational	CD + UC (before/after anti-TNF)	↑ in CD, ↑↑ UC; ↓ after anti-TNF; reflects activity/response
Gunawan et al. [94] Cross-sectional exploratory	CD + UC (serum, urine, stool)	↑ urinary chemerin with ↑ fecal calprotectin; may reflect mucosal inflammation
Tian et al. [8] Meta-analysis	CD + UC (10 case–control studies, 2717 participants)	↑ chemerin levels in IBD; ↑ ↑ in active disease; proposed as a potential biomarker
Baram et al. [97] Cross-sectional, case–control study	IBS (Rome III; subtyped D, C, A)	↑ in IBS (↑↑ IBS-D); correlates with disease symptoms and stress
Roczniak et al. [98] Cross-sectional, case–control study	Pediatric IBS	↑ Chemerin (↓ omentin-1) in IBS; Chemerin ≥232.8 ng/mL: ↓ sensitivity, ↑ specificity for IBS

Arrows indicate direction of change (↑ increase, ↓ decrease).

## Data Availability

Data are contained within the article.

## Abbreviations

AGS	Human gastric adenocarcinoma cell line
ALT	Alanine aminotransferase
AP	Alkaline phosphatase
AST	Aspartate aminotransferase
AUC	Area under the curve
BMI	Body mass index
CA19-9	Carbohydrate antigen 19-9
CCRL2	C-C chemokine receptor-like 2
CD	Crohn’s disease
CEA	Carcinoembryonic antigen
CLIF-SOFA	Chronic Liver Failure–Sequential Organ Failure Assessment
CMKLR1	Chemokine-like receptor 1 (ChemR23)
CRC	Colorectal cancer
CXCL1/2	C-X-C motif chemokine ligand 1/2
DAA	Direct-acting antivirals
DSS	Dextran sodium sulfate
EBV	Epstein–Barr virus
EGR1	Early growth response 1
EMT	Epithelial–mesenchymal transition
EPIC	European Prospective Investigation into Cancer and Nutrition
ERCP	Endoscopic retrograde cholangiopancreatography
ERK	Extracellular signal–regulated kinase
ERK1/2	Extracellular signal–regulated kinases 1 and 2
FAP	Fibroblast activation protein
FIZZ1	Found in inflammatory zone 1 (Resistin-like molecule alpha, RELMα)
FOS	FBJ murine osteosarcoma viral oncogene homolog (c-Fos)
GM-CSF	Granulocyte–macrophage colony-stimulating factor
GPR1	G protein–coupled receptor 1
HBV	Hepatitis B virus
HCC	Hepatocellular carcinoma
HCV	Hepatitis C virus
HLAP	Hyperlipidemia-induced acute pancreatitis
HR	Hazard ratio
IBD	Inflammatory bowel disease
IBS	Irritable bowel syndrome
IFN-γ	Interferon gamma
IL-10	Interleukin-10
IL-17	Interleukin-17
IL-4	Interleukin-4
IL-6	Interleukin-6
INR	International normalized ratio
KATO III	Human gastric cancer cell line KATO III
LC3	Microtubule-associated protein 1 light chain 3
LC3-II	Microtubule-associated protein 1 light chain 3-II
LPO	Lactoperoxidase
LPS	Lipopolysaccharide
MAFLD	Metabolic dysfunction–associated fatty liver disease
MAP	Mean arterial pressure
MAPK	Mitogen-activated protein kinase
MAPKs	Mitogen-activated protein kinases
MASLD	Metabolic dysfunction–associated steatotic liver disease
MDSC	Myeloid-derived suppressor cell
MELD	Model for End-Stage Liver Disease
MKN28	Human gastric cancer cell line MKN28
MMP	Matrix metalloproteinase
MUC2	Mucin 2
NAFLD	Nonalcoholic fatty liver disease
NASH	Nonalcoholic steatohepatitis
NF-κB	Nuclear factor kappa B
OR	Odds ratio
p38	p38 mitogen-activated protein kinase
PCT	Procalcitonin
PD-L1	Programmed death-ligand 1
PEP	Post-ERCP pancreatitis
PI3K	Phosphoinositide 3-kinase
PKC	Protein kinase C
PKM2	Pyruvate kinase M2
PTEN	Phosphatase and tensin homolog
RARRES2	Retinoic acid receptor responder 2 (chemerin)
ROC	Receiver operating characteristic
ROCK	Rho-associated coiled-coil containing protein kinase
RhoA	Ras homolog family member A
SMMC7721	Human hepatoma cell line SMMC-7721
SPP1	Secreted phosphoprotein 1 (osteopontin)
SRF	Serum response factor
STAT6	Signal transducer and activator of transcription 6
TFF3	Trefoil factor 3
TIG2	Tazarotene-induced gene 2 (chemerin)
TIMP	Tissue inhibitor of metalloproteinases
TME	Tumor microenvironment
TNBS	2,4,6-Trinitrobenzene sulfonic acid
TRX1	Thioredoxin 1
UC	Ulcerative colitis
VAT	Visceral adipose tissue
VCL	Vinculin
VEGF	Vascular endothelial growth factor
Ym1	Chitinase-like protein 3 (Ym1/CHI3L3)
hsCRP	High-sensitivity C-reactive protein
